# A Case Report of Seronegative Limbic Encephalitis

**DOI:** 10.7759/cureus.4681

**Published:** 2019-05-16

**Authors:** Maham Munawar, Pulwasha M Iftikhar, Jehanzeb A Khan, Nadir A Syed, Taimur A Syed

**Affiliations:** 1 Internal Medicine, Dow University of Health Sciences, Karachi, PAK; 2 Health Sciences, St. John's University, New York, USA; 3 Internal Medicine, Civil Hospital Karachi, Dow University of Health Sciences, Karachi, PAK; 4 Internal Medicine, South City Hospital, Karachi, PAK; 5 Internal Medicine, Brown University, Providence, USA

**Keywords:** limbic encephalitis, seizures, autoimmune, inflammation, delirium, paraneoplastic, cognitive decline

## Abstract

Most patients with autoimmune encephalitis do not present with well-described symptoms. Demographic data and information regarding co-morbidities could help in diagnosing the underlying disorder, but a definitive diagnosis is made by the result of autoimmune antibodies. Limbic encephalitis (LE), a variant of autoimmune encephalitis, is the inflammation of the limbic system of the brain. The disorder presents with the rapid development of confusion, working memory impairment, mood changes, and often seizures. LE could have paraneoplastic or non-paraneoplastic etiology. We present the case of a 15-year-old girl with seronegative LE, who presented with cognitive decline and seizures. This condition is rare, and therefore poses a great challenge in diagnosis at an early stage.

## Introduction

Limbic encephalitis (LE) was first described by Brievely and Corsellis in 1960 as inflammation of the limbic area, particularly the hippocampus and amygdala, and which could also involve the cerebellum and brainstem [1-2]. It generally presents with amnesia, psychiatric symptoms, an altered level of consciousness, and seizures. This disease generally has two etiologies, paraneoplastic and autoimmune. The paraneoplastic form is associated with certain tumors, including small cell lung cancer (SCLC), germ-cell testicular tumor, breast cancer, Hodgkin’s lymphoma, immature teratoma, and thymoma [3]. In the past few years, significant research has been done on the autoimmune variant of LE and its association with several antibodies, particularly the anti-GAD antibody [4]. However, the diagnosis of the seronegative variant is challenging, and it subsequently leads to a delay in initiating therapy [5]. Here, we present the case of a 15-year-old girl who presented with seronegative limbic encephalitis.

## Case presentation

A 15-year-old girl presented to the emergency department with complaints of febrile illness, cognitive decline, and grand mal epileptic seizures. She had no previous history of viral or bacterial illness, epilepsy, or any other medical condition. On clinical examination, her blood pressure was 90/60 mm Hg, her pulse was 80/bpm, and her temperature was 101 F. The mini mental state examination showed a score of 20/30, and she also had tonic contractions and clonic jerks.

The initial laboratory investigation revealed a white blood cell (WBC) count of 4.9x103 mm​³ a platelet count of 248x102 mm​³​, and Hb level of 9.8g/dl. The serum electrolytes, renal function tests, and liver function tests were within the normal range. Serology for hepatitis B and hepatitis C turned out to be non-reactive. Erythrocyte sedimentation rate was 30mm/hour. Lumbar puncture revealed a clear cerebrospinal fluid (CSF) with a lymphocyte count of fewer than 5 cells/ml, proteins 21mg/dl, and glucose of 58mg/dl. The autoimmune workup exhibited negative anti-nuclear antibody, anti-neutrophilic cytoplasmic antibody, rheumatoid factor, and a negative autoimmune encephalitis mosaic 6, which includes AMPA, CASPR2, DPPX, GABA, LGI1, NMDA. Chest X-ray and abdominal X-ray findings were normal. An electroencephalogram (EEG) recording revealed generalized epileptic activity in the cortex. Magnetic resonance imaging (MRI) and positron emission tomography (PET) demonstrated hypoperfusion in the hippocampal region, which confirmed the diagnosis of non-paraneoplastic limbic encephalitis.

During her hospital visit, she was treated symptomatically for an acute episode of delirium, and her seizures were controlled through IV diazepam. After a confirmed diagnosis of limbic encephalitis, she received an initial course of intravenous immunoglobulin (IVIG) at 0.4 gram/kg of bodyweight for five days, followed by once weekly for four weeks. She was also given IV methylprednisolone for a week. She was discharged on 45 mg of oral prednisolone, which was later tapered off, and the anti-epileptic drugs were continued for a year until her EEG became normal. Magnetic resonance imaging performed after the treatment showed complete resolution of the inflammation in the limbic system of the brain. The patient improved significantly after completion of treatment.

## Discussion

 ​Autoimmune encephalitis is a spectrum of neurological diseases characterized by the formation of autoantibodies of autoimmune or paraneoplastic origin against neuronal surfaces, synaptic antigens, or intracellular proteins [6]. It has been classified into various types on the basis of the anatomical sites or the fundamental etiological process. One such variant of this spectrum is LE, which distinctively involves the limbic system. The underlying pathophysiology of LE could be broadly classified into autoimmune or paraneoplastic. However, it is not uncommon for it to present with non-typical etiologies including the non-paraneoplastic and the seronegative variant [7]. LE shows extensive diversity in symptoms ranging from delusions, hallucinations, irritability, aggression, subacute confusion, memory impairment, and seizures. The medio-temporal lobe is the usual site of origin of seizures, which later develops into secondary generalized seizures resistive to a wide class of anti-epileptic drugs [8-9]. LE shows slight male dominance with the ratio of 2:1 and patients of age above 40 years are commonly affected.

The disease is associated with a multitudinous amount of antibodies responsible for the respective symptom. A timely diagnosis of all these antibodies leads to a better prognosis [10]. Diagnostic criteria for LE includes: neuropsychiatric disorders, history of malignancy within a four-year period, exclusion of symptoms associated with cancer, findings of CSF suggestive of ongoing inflammation, characteristic findings in histopathological examinations, and typical abnormality in EEG and MRI [11]. In 2011, Eric Lancaster elaborately studied the treatment options for the two main forms of LE [12]. However, the treatment of the seronegative form was not emphasized upon. In our case, the patient was treated with the same protocol as seropositive autoimmune LE with IVIG and methylprednisolone. The steroids were gradually tapered off, and an MRI performed after this regime showed complete resolution of the inflammation, and the health of the patient improved significantly. Similar to what was discussed by Dalmau, the seizures of our patient were aggressively treated with IV diazepam during the acute illness, and she continued to receive oral antiepileptic drugs, which were discontinued after a year of normal EEG activity without any need for long-term anti-epileptic drug therapy [13]. Another important aspect related to the management was the prompt treatment. ​Robert B. Darnell, in his study, highlighted the importance of prompt treatment, starting even while waiting for the confirmation of diagnosis, and the categorization of the antibody screen to achieve the best possible prognostic outcome and to prevent permanent sequela [14]. LE, defined by Brierely and Corsellis in 1960, was initially identified as a paraneoplastic disorder [1]. However, recently, the autoimmune version has been studied in more detail. As described by Ahmad SAB, the autoimmune variant of LE still poses a significant challenge during diagnosis as the typical antibody screen does not look for the antibodies associated with this diagnosis [5].

Furthermore, the seronegative subcategory of autoimmune LE further makes the diagnosis hard since it does not seem to be associated with the antibodies including the autoimmune encephalitis mosaic 6 screen [5]. Our patient met the diagnostic criteria of autoimmune LE, as mentioned above, however, after thorough screening, she was not found to have any neoplastic condition and her autoimmune screen tested negative for ANA, ASMA, and AMA. The autoimmune encephalitis mosaic 6 screen failed to show antibodies directed against AMPA, CASPR2, DPPX, GABA, LG1, and NMDA. This case report reinforces the suggestion of Robert B. Darnel that there are probably more antibodies that we have yet discovered [14]. It seconds the need for new and more sensitive techniques, such as those described by Beau MA and colleagues​ t​o find antibodies other than the ones previously identified, which might be associated with this condition [15]. 

## Conclusions

Limbic encephalitis (LE) is an established cause of neurocognitive decline and psychiatric symptoms that are treatable if identified early; delay in diagnosis can leave the patient with permanent losses. MRI plays a pivotal role in establishing a diagnosis, where it shows hyper-intensive lesions, usually in the medial part of the temporal lobes. Patients diagnosed with LE should undergo oncological diagnostics and observatory studies to look for a neoplastic or a paraneoplastic etiology, and they should also undertake the autoimmune screen to look for a possible autoimmune etiology. New and more sensitive techniques should be used to identify unrecognized antibodies that might be associated with those cases of autoimmune encephalitis that are currently considered seronegative. Prompt treatment should be initiated to prevent long-term sequela and to allow a speedy recovery. Treatment includes resection of the underlying tumor, steroids, IVIG, and cyclophosphamide and rituximab in resistant cases. 
